# Sharing success – understanding barriers and enablers to secondary prophylaxis delivery for rheumatic fever and rheumatic heart disease

**DOI:** 10.1186/s12872-016-0344-x

**Published:** 2016-08-31

**Authors:** Jennifer Chamberlain-Salaun, Jane Mills, Priya M. Kevat, Marc G. W. Rémond, Graeme P. Maguire

**Affiliations:** 1 School of Health and Biomedical Sciences, RMIT University, PO Box 71, Bundoora, VIC 3083 Australia; 2 James Cook University, College of Medicine and Dentistry, PO Box 6811, Cairns, QLD 4870 Australia; 3 Baker IDI, PO Box 6492, Melbourne, VIC 3004 Australia; 4 Present Address: Royal Children’s Hospital Melbourne, 50 Flemington Road, Parkville, VIC 3052 Australia

**Keywords:** Rheumatic fever, Rheumatic heart disease, Secondary prophylaxis, Benzathine penicillin, Penicillin

## Abstract

**Background:**

Rheumatic fever (RF) and rheumatic heart disease (RHD) cause considerable morbidity and mortality amongst Australian Aboriginal and Torres Strait Islander populations. Secondary antibiotic prophylaxis in the form of 4-weekly benzathine penicillin injections is the mainstay of control programs. Evidence suggests, however, that delivery rates of such prophylaxis are poor.

**Methods:**

This qualitative study used semi-structured interviews with patients, parents/care givers and health professionals, to explore the enablers of and barriers to the uptake of secondary prophylaxis. Data from participant interviews (with 11 patients/carers and 11 health practitioners) conducted in four far north Queensland sites were analyzed using the method of constant comparative analysis.

**Results:**

Deficits in registration and recall systems and pain attributed to injections were identified as barriers to secondary prophylaxis uptake. There were also varying perceptions regarding responsibility for ensuring injection delivery. Enablers of secondary prophylaxis uptake included positive patient-healthcare provider relationships, supporting patient autonomy, education of patients, care givers and healthcare providers, and community-based service delivery.

**Conclusion:**

The study findings provide insights that may facilitate enhancement of secondary prophylaxis delivery systems and thereby improve uptake of secondary prophylaxis for RF/RHD.

## Background

RF is an auto-immune condition caused by earlier infection with group-A streptococcus (GAS), a common bacterium associated with throat and skin infections [1]. RF is associated with fever and inflammation of the skin (rash), joints (arthritis), brain (chorea) and heart (carditis). While most effects of RF are transitory, carditis associated with RF may lead to permanent damage to heart valves. This chronic condition is termed RHD. Severe heart valve damage may necessitate heart surgery with repair or replacement of affected valves.

RF and RHD result in significant preventable morbidity, mortality and health care utilisation [2, 3]. In Australia, RF occurs almost exclusively in Aboriginal and Torres Strait Islander populations, particularly those living in rural and remote northern and central Australia [4]. The burden of RF and RHD in these populations is amongst the highest documented in the world [5, 6]. Indigenous children aged 4 to 15 years are particularly at risk of RF and of subsequently developing RHD [4].

While a single episode of RF can result in permanent damage to the heart valves, RHD is more likely to develop after recurrent episodes of RF [7, 8]. Furthermore, recurrent episodes of RF in an individual who already has RHD are likely to cause further injury to the heart valves thereby increasing severity of disease. Recurrent episodes of RF can be prevented by stopping re-infection with GAS, through the use of regular antibiotics (termed secondary prophylaxis). The most effective method for achieving this is through the use of four-weekly, long-acting intramuscular benzathine penicillin (LAB) injections [3, 9, 10]. Secondary prophylaxis is a core component of RF/RHD management strategies [1].

Despite the demonstrated effectiveness of secondary prophylaxis in preventing recurrent RF [11], delivery can be challenging. A number of studies have shown that in many countries uptake of LAB is less than optimal [12]. We have demonstrated that less than 20 % of individuals in remote Australia who were prescribed secondary prophylaxis for RF/RHD received ≥80 % of scheduled doses in a twelve month period; the median number of doses over the preceding year was only six of a recommended thirteen [13]. These findings imply that many Indigenous Australians with RF/RHD are left at risk of avoidable and progressive heart damage due to suboptimal delivery of LAB secondary antibiotic prophylaxis.

Addressing low uptake of secondary prophylaxis for RF/RHD has been identified as a public health priority in Australia [14]. Nonetheless, understanding the reasons for poor uptake remains limited. A literature review previously conducted by three of the authors highlights a lack of high quality evidence relating to initiatives to improve the delivery of secondary prophylaxis [12]. The identified lack of evidence provided justification for the current study. The aim of this study was to gain a better understanding of the enablers of, and barriers to, the uptake of secondary prophylaxis by Aboriginal and Torres Strait Islander people with RF and/or RHD. Improved understanding of these factors will enable health care organisations and communities to better identify and develop strategies to improve uptake.

## Methods

### Study design

The research team used a qualitative descriptive study design. This design was chosen as it “is especially amenable to obtaining straight and largely unadorned (i.e., minimally theorized or otherwise transformed or spun) answers” [15]. Using a qualitative descriptive study design enabled the researchers to explore and gain understanding of participants’ experiences of secondary prophylaxis and their perspectives on the enablers and barriers to uptake.

### Sample

Participants were snowball sampled through the use of key informants who had contacts or were working in health clinics in one or more of the four far north Queensland communities included in the study. These communities are geographically remote, have predominantly Aboriginal and Torres Strait Islander populations, and have a documented high burden of RF and RHD. Snowball sampling offered a practical method for accessing ‘hard to reach’ populations [16]. Key informants enabled researchers to access study participants in communities where individuals can be highly mobile (frequently moving between communities), where cultural sensitivities may make direct access to potential participants difficult and where there are inconsistencies in staffing due to both high staff turnover and a reliance on external staff delivering outreach services [17]. Initial study participants in each community were asked to recommend other participants from one or more of the following groups:

- Aboriginal and/or Torres Strait Islander individuals who had been prescribed secondary prophylaxis for RF/RHD
- parents/care givers of Aboriginal and/or Torres Strait Islander children prescribed secondary prophylaxis for RF/RHD
- health professionals who provide treatment to the above groups

### Data generation

Data were generated between October 2013 and February 2014 via face-to-face interviews in four communities across the Cape York and Torres Strait regions of far north Queensland, Australia. Interviews followed a semi-structured format guided by a grand tour question, which was modified to suit health professional or patient interviewees, as follows: “Tell me about your experiences of treating/receiving treatment for rheumatic fever and/or rheumatic heart disease?” Interviews were audio-taped and professionally transcribed verbatim. All participants were provided with written information about the study and informed consent was obtained prior to participating in the study.

### Data analysis

Interview transcripts were imported into NVivo for MAC data management software version 10.2.1. Analysis of transcripts was conducted using the method of constant comparative analysis of data to data, data to incident, incident to incident and incident to category [18]. The first five transcripts were open coded by authors JCS and PK, and a code-book developed that was then used to analyze the remainder of the dataset. Codes were then compared with codes and collapsed into high order categories. All authors contributed to this process. This resulted in six key themes, which respectively describe health professionals’ and patients’ perspectives of treating and receiving treatment for RF/RHD.

## Results

A total of 24 participants participated in 22 interviews; one interview was conducted with an adolescent and their care giver and one interview was conducted simultaneously with two health professionals. The audio of two interviews was inaudible and hence only 20 interviews were transcribed; 11 with patients/care givers and 11 with health practitioners.

A number of themes were identified. These are summarized in Table 1 and outlined in more detail below. Participants’ verbatim quotes are used as evidence to support findings in each of the six themes.

**Table 1:** Elements of health care delivery relevant to secondary antibiotic prophylaxis delivery for RF/RHD

1. Case ascertainment/registration/recall systems	
2. Pain of injections	
3. Locus of responsibility (patient/care giver/clinician)	
4. Site of service delivery	
5. Education	
6. Health professional-patient relationships	

### Case ascertainment/registration/recall systems

Ensuring that patients access diagnostic and treatment services for RF/RHD is challenging for health services. Effective disease registers rely on both an efficient and accurate system for identifying patients requiring ongoing follow-up (case ascertainment/registration) and systems for prompting follow-up (reminder/recall). Given that case ascertainment and registration rely on broader health care access and systems, the focus of this study was the processes associated with follow-up and delivery of secondary antibiotic prophylaxis.

The use of reminder/recall systems ensures that patients receive treatment reminders either prior to when treatments are due or, if the service is not provided in the relevant timeframe, when they become overdue. Such reminders can include patient-held reminders (wallet cards, mobile device applications), direct visits to a patient’s homes or school, posting of lists in prominent public spaces (e.g., local store), letters or emails. The health services involved in this study rely on a number of such systems.

In some instances there is duplication in register systems. Thus, some services use a combination of a centralized State based reminder/recall system, regional databases and local databases. The sharing of registration (who requires prophylaxis) and service delivery (when prophylaxis was delivered) information between different systems creates issues in regards to capturing and accurately recording data. On the whole, health services included in the study receive State based patient recall lists from Rheumatic Heart Disease Queensland, which they cross check with regional databases to see which patients are due or overdue for secondary prophylaxis. Once patients receive injections, health professionals update local, regional and State based systems. The following health professional’s recount of the process highlights the complexity of duplicate systems:

*Well, RHD Australia, they send out a bicillin recall list every first week of each month. I print that off the computer and then with that information I’ve got to go and sit on MD3 [regional database] and get [the local] bicillin recall list. Then I’ve got to update the information off MD3 onto RHD Australia’s recall list and from there I’ve got to go and sit on my computer and update my system. […] Once it’s updated – the RHD Australia recall list, then I fax it back to them or I can scan it and email it back to them. (P11)*

Interviews with health professionals from a health service in another community further highlight the complexities of duplicate systems. Health professionals explained that they use the centralized State-based reminder/recall register as “a kind of reminder” (P16), the health service district’s electronic patient information system for managing “all the overdue rheumatic heart disease” (P16), and a local system, comprising both a list of overdue patients on a wall-chart and a staff diary, to record visits to overdue clients:

*We go out and give them [overdue patients] invites. We go to home visits if they haven’t turned up and we’re continually doing weekly invites. We make sure that it’s in the diary that’s kept for all staff as a reminder of who’s going out. (P17)*

### Pain of injections

Interview data presents a range of strategies used to address the pain associated with LAB injections and suggests that pain remains a deterrent to uptake. Strategies used to minimize pain include the use of paracetamol-based pain relief, ice-packs, topical anesthetic creams and warming the needle. During interview one health professional also referred to the potential use of analgesic gas, although, at the time of interview this had not yet been implemented. The health professional explained:

*We’ve got an entonox cylinder and a circuit. We haven’t yet got a workplace instruction […] we’re waiting for that to happen and we’re hoping that – because they use that at the hospital to help improve the – decrease the amount of distress (P14).*

Data relating to pain associated with LAB injections reveals a number of differing perspectives. From the perspective of health professionals, pain is generally considered to be a “major barrier” (P12) to uptake of LAB. One health professional described the following incident, which he emphasized was “not uncommon” (P12):

*I just had a young lady who is not confirmed rheumatic but she got Strep sores so we do the usual bicillin. She was fine in the room with me, but when she went out to the nurse she took off down the street. (P12)*

Another health professional explained their responsibility for administering LAB injections in a way that minimizes patients’ pain and increases the chance of patients adhering to treatment uptake. As the health professional explained, “the injection itself is one of the most painful we can give and if they [patient] have had a bad experience [with LAB injections] they are less likely to turn up for another one” (P19).

Further to the above comment, one health professional described how health care professionals also witness parents’ anguish at seeing their child in pain and struggle themselves with inflicting pain.

*I think it’s just one of those horrible, painful injections that children don’t like, so their parents find it difficult when their child’s kicking up such a stink about, to bring them in. They don’t want to see them have pain. Even though we know we talk with parents; they understand it’s for their heart, they understand it’s for good. But to try and sit on a child and give a horrible injection is very traumatic to the nurse as well. […] It’s a very horrible thing for staff to go through. (P18)*

While health professionals clearly emphasise pain as a barrier to LAB uptake, it is not so clearly emphasised by patients and parents/carer givers. Interview data suggest that in some instances the issue of pain associated with LAB injections diminishes over time. One mother explained that after seven years of receiving treatment her daughter is now used to the pain.

*She’ll know what to do. She’ll get on the bed and lay there […] She knows, when they ask her, which side she last had. She says, “Yes, I’ll have it on this side”. (P6)*

Similarly, young adult patients explained that after years of receiving treatment they had “got used to [the pain]” (P7).

In contrast, the mother of a 15 year old, who has been receiving treatment for five years, explained that the after-pain of LAB injections remains an issue for her son. “He doesn’t want to go. He doesn’t want to go and most of the time he doesn’t want to go because he complains about the after-pain” (P22). Despite health professionals using ice-packs before treatment, this participant explained that her son experiences pain for an hour after receiving an injection.

### Locus of responsibility

For parents, the pain associated with LAB injections is intrinsically linked to locus of responsibility. The anguish of seeing children in pain, as described above, makes it difficult for parents to assume responsibility for treatment particularly when their child is unwilling to receive it. A health professional explained, “It’s very often extremely difficult for Mums and Dads, or any rellies (relatives) to persuade a child to come in” (P13) to the clinic for treatment. Another health professional explained that when parents are confronted with a child who “is screaming and bucking and says no, well that’s the end of the story” (P18).

Although most parents acknowledge responsibility for their child’s treatment, they do not feel that they can ‘force’ their child into receiving their LAB injections. When discussing prophylaxis, the mother of a four year old explained that for some months her daughter has not wanted to get her injection. The participant added, “I can’t force her to go to [the clinic to] have it if she doesn’t want it. […] If I try to take her she’ll just go mad” (P1). Another mother explained how she supports her reluctant adolescent son to receive timely treatment. “I can’t really force him to go but if I say “Okay come on, let’s go”, then he’ll move. But I have to be there” (P22). Finding ways to support children and adolescents to willingly receive treatment is preferable to using the physical force which one mother described: “When I’d take him up there [the clinic] you’d have to have three or four nurses to hold him down” (P4). The participant went on to explain that three years after being diagnosed, her 11 year old son “is really good at it now” (P4). But that was only after she “snapped […] and cried at the same time” and explained to him that she did not want him to die.

Understanding the gravity of RF/RHD means that some children, even from a young age, recognise the importance of receiving treatment. After his mother 'snapped', the 11 year old boy referred to above understood the necessity of LAB injections and now assumes some of the responsibility for his prophylaxis. His mother explained that now her son and his 12 year old sibling, who also has RHD, are the ones that remind her “most of the time” (P4) when they are due for their injections. Similarly the mother of a 10 year old child explained how her daughter ‘knows’ when she has missed an injection. “She’ll say to me, “Mum, I didn’t get my injection this month”” (P6).

Interview data also highlight fundamental differences in individual health professionals’ philosophical positions regarding who is responsible for patients’ treatment. Some health professionals “believe that [their] duty of care is to the child” (P13) or to the adult patient; while there are other health professionals who consider that adherence to LAB treatment is the patient’s responsibility, or in the case of children and adolescents, the responsibility of parents/care givers.

Although health professionals recognize their duty of care in providing treatment, some health professionals believe that “[patients’] need to take up the responsibility” (P11) for their treatment. This belief was reiterated by one health professional participant who was emphatic that the patient’s “health is my concern, not my responsibility” (P20). The same health professional added:

*As a parent, my child’s health is my responsibility. […] An adult - it’s [their] choice. […] Yes I know it [LAB injection] hurts but it’s your health, your responsibility in the end. We have to stop babying people and let people make informed decisions. (P20)*

### Site of service delivery and facilitating access

While the locus of responsibility outlined above related to who prompted patients to present for prophylaxis, and how they did this, this theme did not cover where the treatment was physically provided. Although a number of alternate locations for service delivery were mentioned in study interviews, the predominant site for delivery of prophylaxis remains the primary health care service/clinic.

An example of a non-clinic based service model was outlined by one health professional who has previously worked in the adjacent jurisdiction of the Northern Territory. They explained the approach used there:

*We did not wait for the community to come in, we went out and we ticked people off the register as we gave it [LAB injections] to them wherever it was most convenient for them. […] Sometimes that was in the back of a car, we used to give their bicillin. It was extremely well accepted. (P13)*

In the Northern Territory, delivering treatment in some communities is often linked to the full-moon cycle. While this is longer than the recommended 28 day interval for LAB delivery, the longer time frame (and theoretical increased risk of having a recurrent episode of RF) is rationalized by a belief that this program enhances patient-mediated recall. Nonetheless, it seems this approach is used more to prompt clinical staff rather than to facilitate patient self-presentation.

*All the clinics had the full moon calendar and off they go every month. [Nurses go] out with the health worker and some community workers and jab as many people as possible. (P12)*

The health professional quoted above considers the full-moon approach “a little bit paternalistic” but added that it results in “about a 90 % success rate” (P12). For this health professional, the challenge of LAB uptake is finding a balance between paternalistic approaches, which result in higher uptake, and a “self-empowerment through education approach” (P12), which he estimated results in uptake rates of approximately 60–70 %.

There is no evidence in the interview data that the full moon calendar approach is used in far north Queensland communities included in this study. However, for some health professionals, arriving unannounced at patients’ homes is an option to ensure patients receive timely prophylaxis, as the following health professional explained:

*Do it at home, if it has to come to that. That’s the only way you’ll get them. If you don’t get them you’ll just have to keep pounding and pounding until they are saying, “I give up, come on, let’s go up”. (P16)*

Although it is often health professionals who instigate delivery of treatment in the community as a mechanism for enhancing recall, some patients, particularly children, also reported preferring to receive prophylaxis in their own homes. One mother organizes for her daughter to receive treatment at home, to counteract her daughter’s unwillingness to receive LAB injections.

*A couple of times I rang them [health professionals] to come out. But most of the time they just normally come out and give it [LAB injection] because they know what she’s like (P1).*

Health professionals are also alert to opportunities of ‘catching’ patients who are overdue for their prophylaxis when they present to the health service for other reasons. The following comments provide examples of opportunistic delivery:

*I had one lot one day, the police brought him in because he’d fallen over and hurt himself at football. What was - he’s about 10? He hadn’t had his injection for months and I said to him, “Do you want to have it?” and he said, “Yes, I will.” His mate held his hand while he had his needle (P20).*

*When they’re coming in for a cold or something else and I’m seeing them, I say, “Look, your injection’s due. You happy for me to give it?” (P19)*

In addition to opportunistic delivery, an additional strategy for enhancing delivery is providing the ancillary services that enhance health service access. An identified key component of such support is transport to and from the health service. One clinical service included in this study supports a community bus that has scheduled hourly services to transport patients for appointments. Other clinics organize individual patient transfers to and from the clinic, particularly for patients who are at risk of not attending appointments. One health professional described a range of situations in which patients are provided individual transport:

*…like with the really dysfunctional families and if the children are quite young, we would go out and get them if necessary, particularly when there are other factors why they might not be turning up. So if it’s really, really hot or if it’s just torrentially raining, you can’t expect them to sort of walk up with young children, particularly if they live on [the other side] of town. We’d go and get them. You know, just offer a little assistance like that. (P21)*

### Education

In order for patients and care givers to make informed decisions regarding LAB prophylaxis it is necessary for them to have access to information about RF/RHD and to gain an understanding of factors such as symptoms, treatment and the implications of not receiving prophylaxis. Health professionals consider that patient and parent education relating to RF/RHD is important. Although educational material such as patient booklets and electronic resources are available and accessible to clients, health professionals in the study highlight the need to “revisit” (P14) information and educational messages with patients and their families on a regular basis. One health professional was particularly adamant about the need to educate patients and parents:

*I think the parents need to be sat down and have a good talk to about what RHD is or what happens if you don’t have the injections, or what happens if you do get Strep. […] In the community we need to put more education into learning about heart disease. I see it as a must. […]. Parents need to be educated. That’s the only way the kids can be educated; if the parents know. We need to really get in their ears - parents need to bring their kids up here to get their injections. (P15)*

The same health professional also highlighted the lack of public health messages focusing on RF/RHD and a lack of community understanding of the impacts of the disease. During interview the health professional commented on the extent of public health messages about diabetes and sexually transmitted diseases and contrasted this to the absence of RF/RHD messages, which he believes should be equally as important. “If we can go out there and talk about STIs or diabetes or renal disease out in the open we should start talking about RHD” (P15). The health professional further commented on what he perceives as a key underlying issue:

*in our community, if someone dies of RHD the community doesn’t see it as a problem or a medical issue; they blame other things outside of that. But that makes it difficult when you’re trying to get the word out there to people in the community. […] We need to stand up and talk and get that out there and tell them that actually a serious thing is happening. (P15)*

Patients do not necessarily make a connection between some of the symptoms of RF, such as sore throats or joint pain, and the disease. Providing patients with education is a means of patient “self-empowerment” (P12) and health professionals are well positioned to support patient education. For health professionals to be able to educate and influence patients, they themselves need to be educated about RF/RHD. One health professional explained that when she started working in the community she “didn’t even know the difference between rheumatic fever and rheumatic heart disease” (P11). The opportunity to attend training workshops enabled this health professional to learn more about these conditions and to understand “the importance of why these people are having injections” (P11). While health professionals are typically committed to patient education, there are some who believe that this is subsidiary to the issue of personal responsibility and locus of responsibility outlined above: “unless people take ownership of what it is – that goes for any disease, any medication – all the education in the world won’t do anything” (P18).

### Health professional-patient relationships

Health professionals, who establish trusting relationships with patients, particularly children, feel that they are able to influence prophylaxis uptake. Establishing relationships, however, is noted to be dependent on continuity of health professionals, which in remote communities is not always possible, as the following health professional’s comment highlights:

*there is such a turnover of staff in places like this that they [patients] don’t get a chance to get to know you. People don’t come to these places and stay long. […] Knowing the staff makes a difference […] the continuity of the face, because they [patients] start to trust you. They’re comfortable coming to [someone they know] (P20).*

Having the same health professional administer LAB injections each time also enables patients to “know the technique” (P21) that the health professional uses. Being familiar with a health professional’s technique and trusting the health professional enables a better patient treatment experience, for an otherwise “horrible, painful injection” (P18). When health professionals are able to take the time to establish relationships with patients “it’s easier for the patient to approach the clinic to have their injection – they can see a friendly face” (P18). Supporting the health professional’s remark was a comment by a patient, who named the particular health professional she prefers; “I like it when […] gives it. She’s friendly and she does it gently” (P9).

Creating rapport with a patient, particularly a child, by visiting them at home and administering the LAB injection in a setting where they feel comfortable is conducive to ensuring timely uptake of treatment. The positive outcomes of establishing trusting health professional-patient relationships are highlighted in the following comments:

*one of the nurses here who has actually established a really good relationship with a nine-year-old who was – who initially it took two hours to convince to have her bicillin for her confirmed rheumatic heart. Now, just whenever [the nurse] visits her at home, she happily rolls over and lets her give it to her without any complaints. So, it’s definitely a good time and explanation and relationship development on all of that. (P12)*

*You know, even if you fail to give the injection the first time, you go and you have a bit of a yarn, and you say what it’s for and get to know them, and particularly say, “Where would you like to have it, have you got a bedroom?” You know, because the first few times I went out, [to the client’s house] I was expected to give it on the couch in front of everybody, including the dogs. I said, no, this is not okay, and kids need privacy too. So, I think really it’s that rapport of just getting to know the children particularly. (P13)*

Although some health professionals and patients concede that establishing a relationship between them supports the experience of receiving LAB injections, one patient explained that her relationship with health professionals administering treatment was of no importance. For this 23 year old, having a good relationship with clinical staff “doesn’t matter”. The participant elaborated, “I just think about my health” (P10). Similarly, another adult patient explained that it “doesn’t matter” (P8) for him which health professional administers his LAB injection. These participants’ comments suggest that establishing health professional-patient relationships is more important when the patient is a child.

## Discussion

This study provides important insights into why systems for secondary prophylaxis delivery for RF/RHD may, or may not, be effective. While findings offer perspectives from patients, families and health professionals in one region of Australia, it is likely the issues highlighted here will be valuable to broader Australian and international audiences including health practitioners and policy makers.

**Fig. 1:**
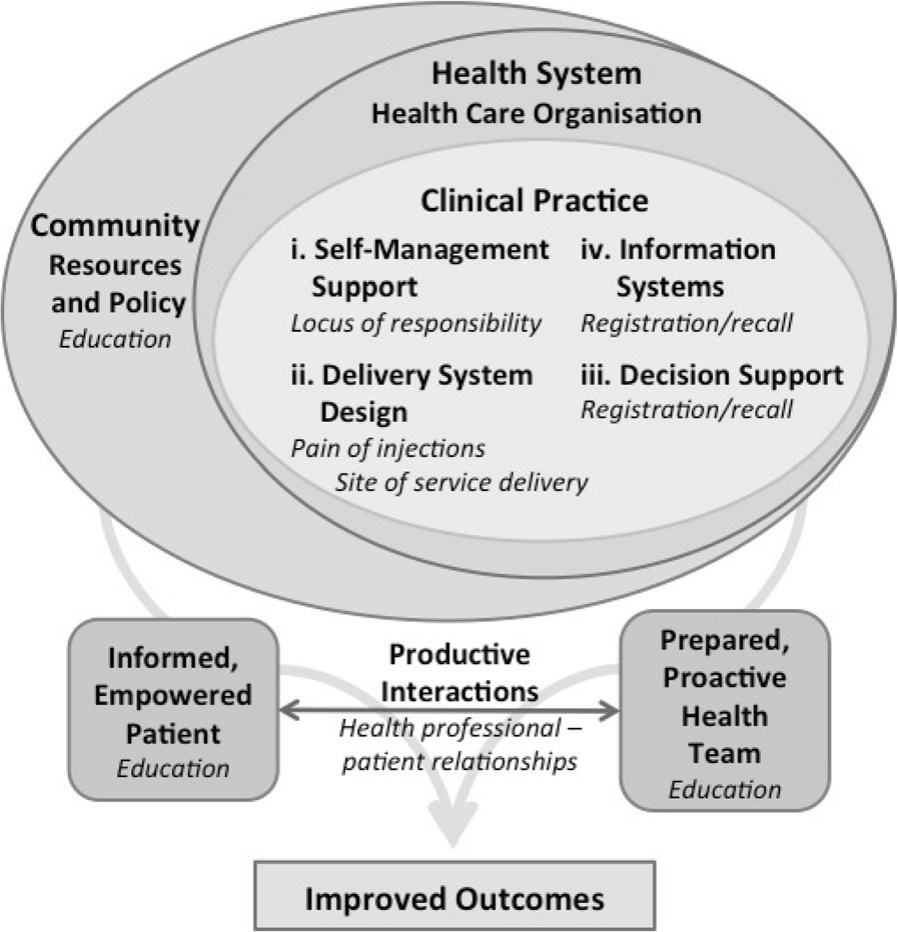
Applying the findings of Sharing Success (in italics) to the Chronic Care Model

In a previously conducted literature review of secondary antibiotic prophylaxis for RF/RHD, Remond et al. [12] examined the delivery of LAB within Wagner’s Chronic Care Model (CCM) framework because of the longevity of prophylaxis required for RF/RHD patients, the low uptake of treatment, and the complexity of delivering health care in this context. Wagner’s CCM encompasses a broad whole-of-system approach that incorporates patient, provider and system-level interventions and has been used as a framework for a broad range of communicable and non-communicable chronic diseases [19]. In Fig. 1, we use this framework to schematically conceptualize and represent our study results. The following discussion contextualizes our study findings within the CCM components of clinical practice, community resources and policy, and productive interactions.

Clinical practice consists of four components: information systems, decision support, delivery system design and self-management support. Case ascertainment and registration and reminder/recall systems are important to supporting the delivery of LAB prophylaxis and constitute information systems and decision support in the CCM. Evidence [20] suggests that providing numerous reminders and using a range of methods to remind patients is effective in improving uptake of regular health treatments.. Findings from our study provide evidence that health providers use a range of initiatives to remind patients about upcoming and overdue LAB prophylaxis. Patients and parents/care givers do acknowledge the usefulness of such reminders, however, the extent to which these strategies are successful in influencing uptake is unclear.

Within the context of information systems, our findings also demonstrate that multiple systems are used at study sites to facilitate LAB registration and recall. This redundancy highlights opportunities for enhancing consistency and simplicity in systems. While study findings do not explain why multiple systems are in place, the requirement of duplicate data entry to a variety of databases, and ongoing use of local registers, suggests that regional RF/RHD register and recall systems do not provide sufficient functionality for local health care services. However, the need for regional databases is evident as failure to track patients across multiple systems and a lack of conformity between health service delivery sites, particularly in remote areas characterized by high staff turnover, are both likely to undermine LAB recall and delivery.

Addressing perceived issues about pain of injections forms part of delivery system design as identified in the CCM. Although study findings clearly demonstrate that health professionals perceive pain as a barrier to LAB uptake, the evidence is not so clear from the patients’/parents’/care givers’ perspectives. The interrelationship of pain with other factors is reflected by Harrington et al. [21], who contend that the pain associated with LAB injections has no simple relationship with uptake of secondary antibiotic prophylaxis. Regardless of the differing perspectives of pain as a barrier to uptake, interview data indicates that reducing the pain associated with delivery of secondary antibiotic prophylaxis delivery is important to patients, parents/care givers and health professionals. Study findings present a range of initiatives used to reduce pain, however whether or not these initiatives are successful is not evident. The limited evidence available in the literature suggests that mixing lidocaine with LAB [22] and using a vibrating device together with a cold pack [23] can reduce the pain associated with administration. Earlier studies also suggest that offering choices to patients may engender perceptions of control that in return can reduce the pain of injections [24]. Thus, in situations where pain is highlighted as an issue impeding delivery of LAB, it may be important for clinicians to enhance perceptions of client control as well as offering pain-relieving options.

Our study findings position locus of responsibility within the CCM component of self-management support. The dynamic tension of responsibility linking patient/care giver and health professional is highlighted. Parents and care givers of patients requiring secondary prophylaxis acknowledge responsibility for ensuring adherence to treatment regimes, yet are reticent to ‘force’ children to receive treatment. Tension similarly exists among health professionals. A previous Australian study [21] found that there is a conflict for health professionals between “providing comprehensive health care and respecting patients’ autonomy”. This finding is reflected in our study, which demonstrates differences in health professionals’ perspectives regarding who is responsible for patients’ treatment. In spite of such tensions, both studies highlight that feeling cared for and nurtured and having a sense of belonging to a health service are important determinants of increased uptake of LAB for patients.

Decisions as to where LAB injections are delivered, constitute delivery system design under the CCM. The importance of local health service delivery reaching into the community is highlighted in this and previous studies [25, 26]. In the case of LAB delivery, linking with the community extends beyond stakeholder, care giver and patient engagement to actual physical delivery of care outside the confines of the clinic. The benefit of community-based delivery is supported by the experience of one LAB delivery program in Auckland, New Zealand. This program focused on community nurses delivering LAB injections in schools and demonstrated high levels of delivery ranging from 80 to 100 % of required doses [27].

While providing intramuscular injections of antibiotics in a community, school or home setting may be daunting to an uninitiated health care provider this can, and is, safely achievable. The possibility of anaphylaxis can be of particular concern but the risk is low [28]. Practical considerations including delivering the first dose within the primary health care clinic or hospital, providing appropriate training to staff and having supplemental oxygen and adrenaline available when providing care in the community are usually sufficient to manage any risk of an allergic reaction or syncope/faint.

Education is a key component of improving chronic disease care and can be envisaged as falling under a number of components of the CCM: community resources and policy, informed empowered patients, and prepared proactive health team. While study results identify education as a key factor in LAB delivery, interviews highlight a diverse range of target audiences including patients, parents/care givers and health professionals. Often the initial target for health education is care givers. As patients become older and develop greater autonomy, the focus of education transitions to the patients themselves. Given many patients requiring LAB prophylaxis in this study are children, it is not surprising that much of the commentary relates to health professionals educating parents/care givers. Educating care givers supports better understanding of the importance of prophylaxis and encourages them to take control of their childrens’ health. Nonetheless many parents/care givers noted that their role is to utilize educational information to in turn educate and influence their child’s behavior. Given that LAB prophylaxis delivery must be continued into adolescence and beyond, focusing only on care givers fails to address this important stage of transition between child-care giver dependency and adulthood. While this is obviously important, how adolescents’ perceptions and needs differed regarding LAB was not captured in this study. Given this remains a high-risk period for both RF recurrence and RHD development and progression, this should be a particular focus for future study.

Health professional-patient relations (productive interactions in the CCM) are a fundamental element of health care delivery. How relations might be enhanced, particularly in linguistically and culturally diverse settings exemplified by remote Indigenous Australian communities, is complex. Key elements are likely to include community perceptions of ownership and control of local health care services. A previous Canadian study by Chandler and Lalonde [26] demonstrates the importance of cultural continuity and community ownership and control on patient mental health outcomes. The importance of such ownership and engagement, as exemplified by formal Aboriginal community-controlled health organizations or less formal engagements between health services and communities, was highlighted in the study and is likely to be similarly important in enhancing LAB uptake.

**Table:** 

Recommendations arising from this study include:
Further investigation regarding what functionality would be required from a single centralized registration and reminder/recall system that could support jurisdictional and local health service needs.
Developing and implementing patient education initiatives, which focus on patients transitioning from child-care giver dependency to adulthood.
Implementing pain-relieving strategies when delivering LAB, where considered necessary.
Consideration of community-based delivery of LAB and/or provision of community transport, where appropriate.

### Study limitations

The use of snowball sampling in the study meant that the size of the sample was reliant on the key informants and study participants to recommend and approach subsequent participants. It is therefore difficult for the researchers to ascertain whether study data is indicative of enablers and barriers to the uptake of secondary prophylaxis in other remote communities in Australia. Study findings, however, highlight issues that will be valuable to broader Australian and international audiences, including policy makers.

## Conclusions

Delivery of secondary antibiotic prophylaxis for RF/RHD remains a priority for reducing the impact of this preventable cause of heart disease both in Australia and globally. This study highlights that a number of barriers persist to achieving optimal uptake of LAB in remote Indigenous Australian communities including deficits in existing registration and recall systems, the pain of injections, and varying perceptions of locus of responsibility. It also highlights a number of enablers to improving uptake including: supporting patient autonomy; education of patients, care givers and healthcare professionals; positive patient-health professional relationships; and supporting systems for community-based outreach and service delivery.

## Abbreviations

CCM: Chronic Care Model
GAS: Group-A streptococcus
LAB: Long-acting intramuscular benzathine penicillin
RF: Rheumatic fever
RHD: Rheumatic heart disease

## References

[1] RHDAustralia (ARF/RHD writing group), National Heart Foundation of Australia and the Cardiac Society of Australia and New Zealand (2012). Australian guideline for prevention, diagnosis and management of acute rheumatic fever and rheumatic heart disease (2nd edition).

[2] Carapetis JR, Steer AC, Mulholland EK, Weber M (2005). The global burden of group A streptococcal diseases. Lancet Infect Dis.

[3] Marijon E, Mirabel M, Celermajer D, Jouven X (2012). Rheumatic heart disease. Lancet.

[4] Field B, AIHW (2004). Rheumatic heart disease: all but forgotten except among Aboriginal and Torres Strait Islander peoples.

[5] Carapetis JR, Currie BJ (1999). Mortality due to acute rheumatic fever and rheumatic heart disease in the Northern Territory: a preventable cause of death in aboriginal people. Aust N Z J Public Health.

[6] Carapetis JR, Wolff DR, Currie BJ (1996). Acute rheumatic fever and rheumatic heart disease in the top end of Australia’s Northern Territory. Med J Aust.

[7] Meira ZM, Goulart EM, Colosimo EA, Mota CC (2005). Long term follow up of rheumatic fever and predictors of severe rheumatic valvar disease in Brazilian children and adolescents. Heart.

[8] Carapetis JR, Kilburn CJ, MacDonald KT, Walker AR, Currie BJ (1997). Ten-year follow up of a cohort with rheumatic heart disease (RHD). Aust N Z J Med.

[9] Expert consultation on rheumatic fever and rheumatic heart disease. Report of a WHO expert consultation on rheumatic fever and rheumatic heart disease 29 October–1 November 2001. Geneva: World Health Organisation; 2001.

[10] Manyemba J, Mayosi BM (2002). Penicillin for secondary prevention of rheumatic fever. Cochrane Database Syst Rev.

[11] World Health Organization. Rheumatic fever and rheumatic heart disease: report of a WHO expert consultation, Geneva, 29 October - 1 November 2001. WHO technical report series 923. 2004. 15382606

[12] Remond M, Coyle M, Mills J, Maguire G (2016). Approaches to improving adherence to secondary prophylaxis for rheumatic fever and rheumatic heart disease: a literature review with a global perspective. Cardiol Rev.

[13] Remond M, Severin K, Hodder Y, Martin J, Nelson C, Atkinson D (2013). Variability in disease burden and management of rheumatic fever and rheumatic heart disease in two regions of tropical Australia. Intern Med J.

[14] Remond M, Wheaton G, Walsh W, Prior D, Maguire G (2012). Acute rheumatic fever and rheumatic heart disease-priorities in prevention, diagnosis and management. A report of the CSANZ Indigenous Cardiovascular Health Conference, Alice Springs 2011. Heart Lung Circ.

[15] Sandelowski M (2000). Whatever happened to qualitative description?. Res Nurs Health.

[16] Heckathorn D (2011). Snowball versus respondent-driven sampling. Sociol Methodol.

[17] Elissa S, Lee R, Binns P, Garstone G, McDonald M (2005). Assessment of a register-based rheumatic heart disease secondary prevention program in an Australian Aboriginal community. Aust N Z J Public Health.

[18] Mills J, Birks M (2014). Qualitative methodology: a practical guide.

[19] Wagner EH, Austin BT, Davis C, Hindmarsh M, Schaefer J, Bonomi A (2001). Improving chronic illness care: translating evidence into action. Health Aff (Millwood).

[20] Jacobson Vann JC, Szilagyi P. Patient reminder and recall systems to improve immunization rates. Cochrane Database Syst Rev. 2005;(3):Art. No.: CD003941. doi:10.1002/14651858.CD003941.pub2. 10.1002/14651858.CD003941.pub2 PMC6485483 16034918

[21] Harrington Z, Thomas DP, Currie BJ, Bulkanhawuy J (2006). Challenging perceptions of non-compliance with rheumatic fever prophylaxis in a remote Aboriginal community. Med J Aust.

[22] Amir J, Ginat S, Cohen YH, Marcus TE, Keller N, Varsano I (1998). Lidocaine as a diluent for administration of benzathine penicillin G. Pediatr Infect Dis J.

[23] Russell K, Nicholson R, Naidu R (2014). Reducing the pain of intramuscular benzathine penicillin injections in the rheumatic fever population of Counties Manukau District Health Board. J Paediatr Child Health.

[24] Weinstein P, Raadal M, Naidu S, Yoshida T, Kvale G, Milgrom P (2003). A videotaped intervention to enhance child control and reduce anxiety of the pain of dental injections. Eur J Paediatr Dent.

[25] White H, Walsh W, Brown A, Riddell T, Tonkin A, Jeremy R (2010). Rheumatic heart disease in Indigenous populations. Heart Lung Circ.

[26] Chandler M, Lalonde C. Cultural continuity as a protective factor against suicide in First Nations youth. Horizons --A Special Issue on Aboriginal Youth, Hope or Heartbreak: Aboriginal Youth and Canada’s Future. 2008;10(1):68–72.

[27] Grayson S, Horsburgh M, Lennon D (2006). An Auckland regional audit of the nurse-led rheumatic fever secondary prophylaxis programme. N Z Med J.

[28] International Rheumatic Fever Study Group (1991). Allergic reactions to long-term benzathine penicillin prophylaxis for rheumatic fever. Lancet.

